# Impact of Diagnostic Delay on Disease Course in Pediatric- versus Adult-Onset Patients with Ulcerative Colitis: Data from the Swiss IBD Cohort

**DOI:** 10.1159/000520995

**Published:** 2021-11-18

**Authors:** Alain M. Schoepfer, Vu Dang Chau Tran, Jean-Benoit Rossel, Christiane Sokollik, Johannes Spalinger, Ekaterina Safroneeva, Thea von Graffenried, Sébastien Godat, Dieter Hahnloser, Stephan R. Vavricka, Christian Braegger, Andreas Nydegger

**Affiliations:** ^a^Division of Gastroenterology and Hepatology, Centre Hospitalier Universitaire Vaudois (CHUV) and University of Lausanne, Lausanne, Switzerland; ^b^Division of Pediatric Gastroenterology, Centre Hospitalier Universitaire Vaudois (CHUV) and University of Lausanne, Lausanne, Switzerland; ^c^Clinical Trials Unit, Institute of Social and Preventive Medicine, University of Bern, Bern, Switzerland; ^d^Division of Pediatric Gastroenterology, Hepatology, and Nutrition, University Children's Hospital, University of Bern, Bern, Switzerland; ^e^Division of Pediatric Gastroenterology, Children's Hospital LUKS, Lucerne, Switzerland; ^f^Institute of Social and Preventive Medicine, University of Bern, Bern, Switzerland; ^g^Division of Visceral Surgery, Centre Hospitalier Universitaire Vaudois (CHUV) and University of Lausanne, Lausanne, Switzerland; ^h^Division of Gastroenterology and Hepatology, University Hospital Zurich, Zurich, Switzerland; ^i^Nutrition Research Unit, University Children's Hospital Zurich, Zurich, Switzerland

**Keywords:** Ulcerative colitis, Colectomy, Natural history, Inflammatory bowel disease, Diagnostic delay

## Abstract

**Introduction:**

Given the lack of data, we aimed to assess the impact of the length of diagnostic delay on the natural history of ulcerative colitis (UC) in pediatric (diagnosed <18 years) and adult patients (diagnosed ≥18 years).

**Methods:**

Data from the Swiss Inflammatory Bowel Disease Cohort Study were analyzed. Diagnostic delay was defined as the interval between the first appearance of UC-related symptoms until diagnosis. Logistic regression modeling evaluated the appearance of the following complications in the long term according to the length of diagnostic delay: colonic dysplasia, colorectal cancer, UC-related hospitalization, colectomy, and extraintestinal manifestations (EIMs).

**Results:**

A total of 184 pediatric and 846 adult patients were included. The median diagnostic delay was 4 [IQR 2–7.5] months for the pediatric-onset group and 3 [IQR 2–10] months for the adult-onset group (*p* = 0.873). In both, pediatric- and adult-onset groups, the length of diagnostic delay at UC diagnosis was not associated with colectomy, UC-related hospitalization, colon dysplasia, and colorectal cancer. EIMs were significantly more prevalent at UC diagnosis in the adult-onset group with long diagnostic delay than in the adult-onset group with short diagnostic delay (*p* = 0.022). In the long term, the length of diagnostic delay was associated in the adult-onset group with colorectal dysplasia (*p* = 0.023), EIMs (*p* < 0.001), and more specifically arthritis/arthralgias (*p* < 0.001) and ankylosing spondylitis/sacroiliitis (*p* < 0.001). In the pediatric-onset UC group, the length of diagnostic delay in the long term was associated with arthritis/arthralgias (*p* = 0.017); however, it was not predictive for colectomy and UC-related hospitalization.

**Conclusions:**

As colorectal cancer and EIMs are associated with considerable morbidity and costs, every effort should be made to reduce diagnostic delay in UC patients.

## Introduction

The diagnosis of inflammatory bowel disease (IBD) can be challenging due to an overlap of symptoms, such as chronic abdominal pain and diarrhea, with, for example, irritable bowel syndrome [1]. Our group was the first to systematically evaluate the length of diagnostic delay, defined as the time interval from IBD-related symptoms to IBD diagnosis, in adult patients of the Swiss IBD Cohort [1, 2]. We found that the median diagnostic delay in Crohn's disease (CD) patients was 9 months compared to a median of 4 months for patients with ulcerative colitis (UC). Of note, 25% of CD patients were diagnosed with a diagnostic delay >24 months compared to 25% of UC patients who required >12 months for their diagnosis [1]. Age <40 years and isolated ileal disease were identified as risk factors for long diagnostic delay (defined as time >24 months) in CD patients, whereas male gender and use of nonsteroidal anti-inflammatory drugs were identified to be associated with long diagnostic delay (defined as interval >12 months) in adult UC patients [1]. Approximately 25% of all IBD patients are diagnosed during childhood or adolescence [3]. We further evaluated the length of diagnostic delay in the pediatric population of the Swiss IBD Cohort and found a median [IQR] diagnostic delay of 4 [2, 3, 4, 5, 6, 7, 8] months in CD patients compared with 2 [1, 2, 3, 4, 5, 6, 7] months in UC patients [4]. Our group recently published on the impact of diagnostic delay on bowel damage in pediatric- versus adult-onset CD [5]. At diagnosis, adult-onset CD patients more frequently presented with bowel stenosis and bowel surgery than pediatric CD patients [5]. Interestingly, in the long term, the length of diagnostic delay was significantly associated with bowel stenosis, internal fistula, and any complication in the adult-onset CD population; however, no significant association between the length of diagnostic delay and CD-related outcomes could be observed in the pediatric population [5].

Several aspects regarding the natural history of adult UC have been reported by Fumery et al. [6]: First, extraintestinal manifestations (EIMs) and elevated C-reactive protein at diagnosis and at 5 years of follow-up are significantly associated with extensive disease. Second, hospitalization is necessary for almost half of patients at some point during the disease course. The cumulative probabilities for UC-related hospitalization range from 17 to 29%, 29 to 54%, and 39 to 66% at disease duration of 1, 5, and 10 years, respectively, while 10–15% may be hospitalized at UC diagnosis. Third, the need for UC-related surgery and risk of colorectal cancer increases with disease duration [6]. Pediatric onset UC often presents with extensive disease at diagnosis and rapid progression, but fortunately, colorectal cancer in pediatric UC patients younger than 12 years is extremely rare [7]. Several studies [1, 4, 8, 9, 10] reported already on the diagnostic delay in UC patients; however, there are no data on the impact of diagnostic delay on outcomes in pediatric- versus adult-onset UC patients. As such, we aimed to evaluate the relationship between the length of diagnostic delay and development of colonic dysplasia, colorectal cancer, UC-related hospitalization, colectomy, and EIMs in pediatric- and adult-onset UC.

## Materials and Methods

### Study Design

Starting in 2006, the Swiss IBD Cohort Study (SIBDCS) has been including IBD patients from all regions of Switzerland. The SIBDCS is a national prospective cohort study on IBD patients and provides up-to-date information regarding different aspects of IBD in Switzerland for the Swiss and international scientific community, public health authorities, and medical staff [2, 11]. Ethics approval was obtained for the study protocol by the Ethics Committee of Cantons or regions in which patients were included, as well as individual consent of patient and parents. Patients are included in the cohort once diagnosis of CD, UC, or unclassified IBD has been established for at least 4 months [11].

### Methods

The collected data were entered into a Microsoft Access database (Access 2000; Microsoft Switzerland Ltd Liab. Co., Wallisellen, Switzerland) at the datacenter of the SIBDCS and SPIBDCS, which is located at the Center for Primary Care and Public Health (Unisanté) at the University of Lausanne. For this manuscript, the analysis was based on the validated data obtained from IBD patients enrolled into the SIBDCS and SPIBDCS between May 2006 and November 2019. The following data were extracted from physician questionnaires at enrollment and annual follow-up: demographic variables (age, date of birth, age at diagnosis, date of diagnosis, date of first symptoms, date of visit, and gender); UC-related symptoms, as measured by the Modified Truelove and Witts activity index (MTWAI) [12] for pediatric adult patients; and medical items (initial disease location, last disease location, current therapy, EIMs [oral erosions/ulcers, erythema nodosum, pyoderma gangrenosum, arthritis/arthralgia, primary sclerosing cholangitis (PSC), uveitis/iritis, and ankylosing spondylitis/sacroiliitis], NSAID intake, complications [colectomy, UC-related hospitalization, colon dysplasia, colorectal cancer, any complication, and EIM], and past therapies including response and reason for discontinuation).

Diagnostic delay (reported in months) was defined as the time interval between the first appearance of IBD-related symptoms and IBD diagnosis. Diagnostic delay was stratified into the following two time intervals: (1) time from the appearance of first IBD-related symptoms to the first physician visit (family practitioner, pediatrician) and (2) time interval from the first physician visit (due to IBD-related complaints) to the establishment of IBD diagnosis. The term “short diagnostic delay” was used to describe the length of diagnostic delay laying from the 1st to the 3rd quartile, while the term “long diagnostic delay” was used to describe the length of diagnostic delay laying in the 4th quartile. The impact of the length of diagnostic delay of UC-related complications (colectomy, UC-related hospitalization, colon dysplasia, colorectal cancer, any complication, and EIM) was analyzed according to quartiles at UC diagnosis and in the long term.

### Patients

Patients were prospectively included if IBD diagnosis had been established in 2006 or later; moreover, patients with IBD diagnosis in 2005 or earlier were also recruited retrospectively with prospective data capture from 2006 onward. The cutoff used for the pediatric-onset UC patients was <18 years and adult-onset UC patients ≥18 years, as pediatric patients are followed by pediatric gastroenterologists until the age of 18 years. At the time of inclusion, patients underwent a thorough clinical and laboratory assessment. The treating physician completed physician-reported outcomes, whereas patients completed patient-reported outcomes such as questionnaires on quality of life either alone (adult patients) or with the help of a trained study nurse (pediatric patients). Disease location was recorded according to the Montréal classification in adults and the Paris classification in children [13, 14]. Yearly patient-reported questionnaires about quality of life, social impairment, health resource consumption, and symptoms, and yearly physician follow-up questionnaires about treatments and complications were collected.

### Statistical Analysis

Data were retrieved from the database of the SIBDCS at the Center for Primary Care and Public Health (Unisanté) at the University of Lausanne, Switzerland. All statistical analyses were performed by the cohort statistician (JBR) using the statistical program Stata (version 14.2; College Station, TX, USA). Quantitative data distribution was analyzed using Normal Q-Q Plots. Results of quantitative data are presented either as median plus interquartile ranges (for data with non-Gaussian distribution) or mean ± SD and range (for normally distributed data). Categorical data were summarized as the percentage of the group total. For quantitative data, differences in the distribution between two groups were evaluated using either the Wilcoxon-Mann-Whitney rank test (for data with non-Gaussian distribution) or the Student's *t*-test (for normally distributed data). For categorical outcomes, differences in observed frequencies between groups were compared using the χ^2^ test or using the exact Fisher test for groups with a small number of observations (*n* < 5). Time-to-event data were analyzed using the Kaplan-Meier estimator. We assessed the impact of diagnostic delay's length on the following outcomes: colectomy, UC-related hospitalization, colon dysplasia, colorectal cancer, EIMs, and any complication by using the Cox regression model. For the present study, a *p* value <0.05 was considered as statistically significant. A Bonferroni correction was performed in case of multiple testing.

## Results

### Baseline Characteristics

A total of 1,030 UC patients were analyzed, of which 184 (17.9%) were diagnosed in the pediatric age (<18 years) and 846 (82.1%) in adulthood (≥18 years). The demographic characteristics are shown in Table 1. The median disease duration at the latest follow-up in the pediatric-onset group was 9.6 years compared to 12.4 years in the adult-onset group (*p* < 0.001). The percentage of patients with long diagnostic delay, defined as delay >75th percentile, was not different between the pediatric and the adult group. At diagnosis, children more frequently presented with pancolitis than adults (65.5% vs. 38.7%, respectively; *p* < 0.001).

**Fig. 1:**
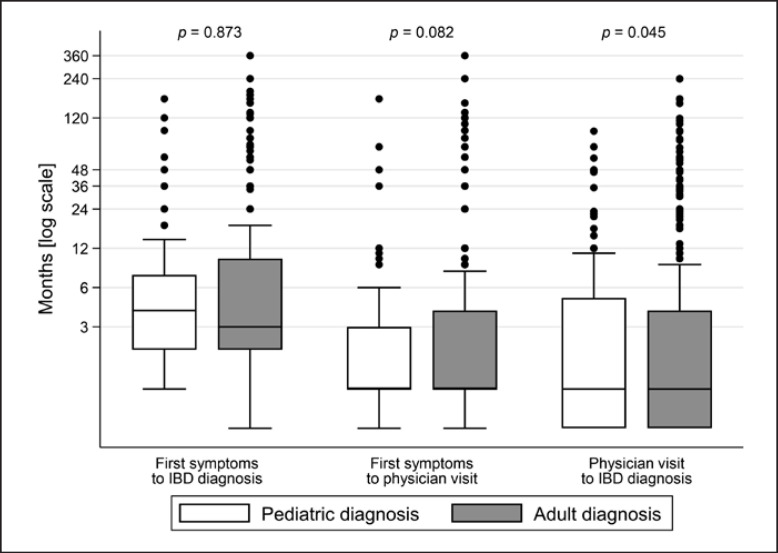
Diagnostic delay (in months) comparing UC patients with adult-onset diagnosis versus patients with pediatric-onset diagnosis. Results are presented by means of box plots. The horizontal line in the box represents the median, whereas the box contains the 25th to the 75th percentile of all values.

The systematic analysis of diagnostic delay in the pediatric and adult UC groups is shown in Table 2 and in Figure 1. The median diagnostic delay was 4 months [IQR 2–7.5] for the pediatric group and 3 [IQR 2–10] months for the adult group (*p* = 0.873). The median time interval between the first UC-related symptoms and the first physician visit due to these symptoms was not different between pediatric- and adult-onset UC patients (median for both groups was 1 month, *p* = 0.082), whereas time from the first physician visit to UC diagnosis was longer in adult-onset UC patients than that in pediatric-onset UC patients (*p* = 0.045).

The prevalence of EIMs in both pediatric- and adult-onset UC patients is shown in Table 3. Arthritis/arthralgia was more frequently diagnosed in adult-onset UC than in pediatric-onset UC patients (36.1% vs. 22.8%; *p* < 0.001), whereas PSC more often occurred in pediatric-onset UC (8.2% vs. 3%; *p* < 0.001). We observed no difference between these groups with respect to oral ulcers, erythema nodosum, pyoderma gangrenosum, uveitis/iritis, and ankylosing spondylitis/sacroiliitis.

The medical therapies ever applied are shown in Table 4. No differences were observed between the two groups regarding the following drug use: any 5-ASA, systemic steroids, any steroids, topical budesonide (rectal foam), immunomodulators (azathioprine/mercaptopurine and methotrexate), anti-TNF agents, vedolizumab, ustekinumab, and tofacitinib. However, topical 5-ASA (enema and suppositories) were used significantly less frequently in the pediatric- than in the adult-onset UC group (46.7% vs. 65.6%; *p* < 0.001).

### Frequency of Complications in the Pediatric- and Adult-Onset Groups at UC Diagnosis

In the pediatric-onset group, the rate of colectomy, UC-related hospitalization, colon dysplasia, colorectal cancer, and EIMs were not different between the group with short diagnostic delay and the group with long diagnostic delay at UC diagnosis Table 5. However, the adult-onset UC group with long diagnostic delay more frequently experienced EIMs at UC diagnosis than the adult-onset group with short diagnostic delay (*p* = 0.022, Table 5; online suppl. Fig. 1; for all online suppl. material, see www.karger.com/doi/10.1159/000520995). There was no difference between the adult-onset group with short versus long diagnostic delay regarding the rate of colectomy, UC-related hospitalization, colon dysplasia, colorectal cancer, and any of the aforementioned complications.

Pediatric-onset UC patients underwent hospitalizations more frequently than adult-onset UC patients (*p* = 0.006, Table 5; online suppl. Fig. 2) and suffered from any complication (defined as combined outcome including colectomy, hospitalization, dysplasia, or colorectal cancer, *p* = 0.009) at UC diagnosis (online suppl. Fig. 2). However, it should be mentioned that at UC diagnosis, no pediatric patient presented with colorectal cancer (short and long diagnostic delays combined).

Comparison of the long-term complication rate was performed between the pediatric- and adult-onset UC groups according to the length of diagnostic delay. We evaluated the occurrence of different long-term complications in the pediatric-onset UC group compared with the adult-onset group using the Cox regression model with hazard ratio (Table 6). We found that the length of diagnostic delay has no impact on the outcomes, such as colectomy, UC-related hospitalization, colorectal cancer, and any complication. In the pediatric-onset group, there were 2 cases with colorectal dysplasia but no case of colorectal cancer. In the adult-onset group, we observed 13 patients with colorectal dysplasias and 8 cases with colorectal cancer. The length of diagnostic delay in the adult population was positively associated with the development of colorectal dysplasia (*p* = 0.023) and any EIM (*p* < 0.001). When analyzing the different EIMs in detail, we found that the length of diagnostic in the adult-onset UC was associated with arthritis/arthralgias (*p* < 0.001) and ankylosing spondylitis/sacroiliitis (*p* < 0.001). Furthermore, when comparing the adult crude HR and adult adjusted HR, there was no association between age and sex and the development of EIM in the adult population. The length of diagnostic delay in the pediatric population was associated with arthritis/arthralgias (*p* < 0.001).

## Discussion

This is the first study to compare the impact of diagnostic delay in patients with pediatric UC onset with that of adult UC onset. A number of study findings are clinically relevant for care of UC patients. First, the length of diagnostic delay was short, with a median of 4 and 3 months in the pediatric- and adult-onset UC cohort, respectively. Second, the length of diagnostic delay at UC diagnosis was not associated with colectomy, UC-related hospitalization, colon dysplasia, and colorectal cancer in both pediatric- and adult-onset groups. However, the length of diagnostic delay was associated with EIMs at the time of UC diagnosis in the adult-onset group. Third, in the long term, the length of diagnostic delay was associated with colorectal dysplasia and EIMs in the adult-onset UC group. In the pediatric and adult population, in the long term, the length of diagnostic delay was associated with arthritis/arthralgia.

Our data are in accordance with results presented by Nguyen et al. [8] who found a median diagnostic delay of 3.1 months in a cohort of 67 UC patients (>16 years of age). In addition, our data compare well to the findings of Rinawi et al. [15] who found a median diagnostic delay of 4 months (IQR 26) in patients not undergoing colectomy and 3 (IQR 1.5–5 months) in patients undergoing colectomy in an Israeli cohort of 188 patients with pediatric UC onset. In a Romanian cohort of 682 UC patients, Zaharie et al. [9] reported a median diagnostic delay of only 1 month, which is considerably shorter than the diagnostic delay we observed in Switzerland. The interval from the first physician visit to UC diagnosis was longer in adult-onset UC patients than that in pediatric-onset UC patients. Zaharie et al. [9] argued that the differential diagnosis of chronic abdominal pain and diarrhea is broader in the adult population, which might contribute to a delayed referral to gastroenterologists. Longer waiting lists to obtain appointments at specialist centers for adult gastroenterology might further contribute to delays compared to the pediatric population in the Swiss healthcare system. In addition, the attitude of the pediatric healthcare specialist might be different toward pediatric patients due to rapid consequences of the disease on growth and parent's worries about their child's well-being. Overall differences in the length of diagnostic delay in UC patients may be related to a multitude of factors such as organizational aspects of the local healthcare system including access to endoscopy and also differences with respect to initial disease severity [16].

When evaluating the impact that the length of diagnostic delay has on complications at the time of UC diagnosis, we found that it was associated with the appearance of EIMs in the adult-onset group. Additionally, pediatric-onset UC patients were more likely to be hospitalized than adults. Our data regarding hospitalization rates are in accordance with data from Fumery et al. [6] who conducted a systematic review on the natural history of adult-onset UC in population-based cohorts (*n* = 15,316 patients) and found that 10–15% of patients needed to be hospitalized at the time of UC diagnosis. In a systematic review on the natural history of pediatric-onset UC in population-based studies, authors from the same group reported on higher frequencies of hospitalization for pediatric-onset than for adult-onset UC, which is again consistent with our results [17]. The EPIMAD registry found that EIMs were present in 7% of pediatric-onset UC patients [18]. In analogy to our cohort, joint manifestations were observed most frequently, followed by skin manifestations, uveitis, and PSC [18]. The rate of colectomy at UC diagnosis in our pediatric-onset and adult-onset cohort was roughly 4% and is thereby comparable with colectomy rates reported in the aforementioned systematic reviews [6, 17].

The long-term natural history of adult-onset UC is characterized by several well-known features. First, left-sided colitis is the most prevalent location, and disease extension can be observed in 10–30% of patients [6]. The majority of patients have a mild-moderate course with most active disease at diagnosis and then varying periods of remission or mild activity [6]. An aggressive disease course is observed in 10–15% of patients, and the cumulative risk of relapse is 70–80% at 10 years [6]. Roughly 50% of patients require UC-related hospitalizations, and the 5-year risk of rehospitalization is about 50% [6]. The 5- and 10-year cumulative risks of colectomy range between 10 and 15%.

The long-term natural history of pediatric-onset UC is also characterized by distinct features. First, most of these patients face disease extension, and roughly two-thirds have pancolitis at the end of follow-up [17]. Fortunately, these patients do not appear to suffer from significant growth retardation or delayed puberty as commonly seen in CD patients [17]. After a follow-up of 10 years, one-half of patients require hospitalizations and 20% of patients require colectomy [17].

As of yet, the impact of the length of diagnostic delay on the long-term natural history of pediatric-onset UC and adult-onset UC was unknown. We found that in the long term, the length of diagnostic delay was associated with the appearance of EIMs both in the pediatric- and adult-onset groups. Arthritis/arthralgia was most frequently observed, followed by oral erosions/ulcers, uveitis/iritis, PSC, erythema nodosum, and pyoderma gangrenosum. In the adult-onset group, the length of diagnostic delay was significantly associated with arthritis/arthralgia, ankylosing spondylitis, and erythema nodosum, while in the pediatric-onset group, a significant relationship was only found with PSC. The difference between the types of EIM found between the adult versus pediatric population might be related to a longer disease duration for the adult group [19]. Up to 50% of IBD patients experience at least one EIM during their disease career, and in up to one-quarter of affected patients, these EIMs may even manifest before IBD diagnosis and might therefore trigger the search for underlying IBD [20, 21]. EIMs are not only responsible for important morbidity but on a social level, they are also associated with relevant additional costs [22]. To the best of our knowledge, this is the first study to demonstrate the significant relationship between the length of diagnostic delay and appearance of EIMs. We conclude that joint efforts have to be launched to further reduce diagnostic delay in UC patients in order to reduce EIM-associated morbidity.

Our study has several strengths and some limitations as well. We present the first prospective data in a national cohort study, evaluating the impact of diagnostic delay in pediatric- versus adult-onset UC patients regarding long-term complications such as colectomy, UC-related hospitalization, colon dysplasia, colorectal cancer, and EIMs. As patients with IBD diagnosed prior to 2006 were retrospectively included, the length of follow-up is long (>10 years). Thanks to long follow-up, we were able to observe the disease course and those complications. As a limitation, we have to acknowledge that the data presented herein are not population-based as 80% of patients are included by gastroenterologists working in hospital. As such, our findings may not apply to the entire IBD population in Switzerland. In addition, the change of clinical practice over time, such as increased use of fecal calprotectin, might influence the length of diagnostic delay and consecutively the disease course. Another limitation is the lack of data collection of combined EIM and the missing information about disease activity at diagnosis.

In summary, we found that the length of diagnostic delay was short in Switzerland and that it was not associated with colectomy, hospitalization, and colorectal dysplasia or cancer at UC diagnosis. In the long term, length the of diagnostic delay was associated to colorectal cancer in the adult-onset group and the appearance of EIMs in both pediatric-onset and adult-onset groups. Given the fact that colorectal cancer and EIMs are associated with considerable morbidity and costs, joint efforts should be launched to further reduce diagnostic delay in UC patients.

## Statement of Ethics

Ethics approval was obtained from the regional Swiss Ethics Committees in which SIBDCS cohort participants were enrolled (Commission d'éthique du Canton de Vaud/Protocol No. 33/06). Written informed consent was obtained from each patient included in the study. The study was conducted in accordance with the World Medical Association Declaration of Helsinki.

## Conflict of Interest Statement

The authors have no conflicts of interest to declare.

## Funding Sources

This work was supported by grants from the Swiss National Science Foundation (33CS30_177523/1 to G.R.). This is an investigator-initiated study; pharmaceutical companies played no role in study design, acquisition, analysis, interpretation, or presentation of the data.

## Author Contributions

A.M.S., V.D.C.T., J.-B.R., E.S., S.G., D.H., and A.N. conceptualized and designed the study. A.M.S., J.-B.S., C.B., S.G., and A.N. collected data. J.-B.R., A.S., E.S., and A.N. performed the statistical analysis. All authors contributed to data interpretation and manuscript writing, and had full access to all data in the study. All authors read and approved the final manuscript.

## Data Availability Statement

The data underlying this article cannot be shared publicly as participating patients of the Swiss IBD Cohort provided their written informed consent only for use of their data in research performed by members of the Swiss IBD Cohort. The data will be shared on reasonable request to the corresponding author.

## Supplementary Material

Supplementary data

Supplementary data

Supplementary data

## Figures and Tables

**Table 1:** Baseline characteristics

UC patients	Pediatric diagnosis (<18 years old)	Adult diagnosis (≥18 years old)	*p* value
Patients, *n* (%)	184 (17.9)	846 (82.1)	−
Gender, *n* (%)			
Male	84 (45.7)	454 (53.7)	0.049
Female	100 (54.3)	392 (46.3)	
Age at diagnosis (median, IQR, range)	14.1, 10.2–15.9, 2.3–17.9	31.8, 25.1–40.8, 18.0–79.6	<0.001
Age at enrollment (median, IQR, range)	16.6, 13.3–24.9, 3.9–54.1	41.4, 32.5–51.8, 18.4–84	<0.001
Disease duration at enrollment (median, IQR, range), yr	2.9, 0.9–11.0, 0.2–39.2	5.4, 1.8–12.4, 0–50.3	0.001
Age at latest follow-up (median, IQR, range)	21.6, 16.4–30, 5.5–62.1	48, 38.3–58.4, 19.6–86.2	<0.001
Disease duration at latest follow-up (median, IQR, range), yr	9.6, 4.2–17.1, 0.3–44.3	12.4, 7.4–19.2, 0.1–54.3	<0.001
Diagnostic delay (median, IQR, range), months	4, 2–7.5, 1–168	3, 2–10, 0–360	0.873
Diagnostic delay categorization, *n* (%)			
Short delay (≤75th percentile)	138 (75)	635 (75.1)	0.987
Long delay (>75th percentile)	46 (25)	211 (24.9)	
Disease location at diagnosis			
E1 (proctitis), *n* (%)	16 (9.7)	193 (25)	<0.001
E2 (left-sided), *n* (%)	41 (24.8)	280 (36.3)	
E3 (pancolitis), *n* (%)	108 (65.5)	299 (38.7)	
Unknown/unclear	19	74	
Disease location at enrollment			
E1 (proctitis), *n* (%)	13 (7.3)	151 (18.2)	<0.001
E2 (left-sided), *n* (%)	51 (28.7)	329 (39.7)	
E3 (pancolitis), *n* (%)	114 (64)	349 (42.1)	
Unknown/unclear	6	17	
Disease location at latest follow-up			
E1 (proctitis), *n* (%)	20 (11)	185 (22.1)	<0.001
E2 (left-sided), *n* (%)	46 (25.3)	336 (40.1)	
E3 (pancolitis), *n* (%)	116 (63.7)	316 (37.8)	
Unknown/unclear	2	9	
Smoking status			
Smoking at enrollment, *n* (%)			
Yes	20 (10.9)	145 (17.1)	0.036
No	164 (89.1)	701 (82.9)	
Smoking at last visit			
Yes, *n* (%)	17 (9.9)	97 (12.9)	0.286
No, *n* (%)	154 (90.1)	654 (87.1)	
Missing	13	95	
Ever smoked, *n* (%)			
Yes	32 (17.4)	285 (33.7)	<0.001
No	152 (82.6)	561 (66.3)	

**Table 2:** Analysis of overall length of diagnostic delay as well as stratification into the two intervals that determine the overall length

Intervals of diagnostic delay	Pediatric UC diagnosis (<18 years old) (*n* = 184)	Adult UC diagnosis (≥18 years old) (*n* = 846)	*p* value
Time between first symptoms and IBD diagnosis (median, IQR, range), months	4, 2–7.5, 1–168	3, 2–10, 0–360	0.873
Time between first symptoms and physician visit (median, IQR, range), months	1, 1–3, 0–168	1, 1–4, 0–360	0.082
Time between physician visit and IBD diagnosis (median, IQR, range), months	1, 0–5, 0–95	1, 0–4, 0–240	0.045

**Table 3:** Frequency of EIMs in both pediatric- and adult-onset UC patients

EIM (ever)	Pediatric UC diagnosis (<18 years old) (*n* = 184), *n* (%)	Adult UC diagnosis (≥18 years old) (*n* = 846), *n* (%)	*p* value
Oral erosions/ulcers	19 (10.3)	48 (5.7)	0.020
Erythema nodosum	4 (2.2)	31 (3.7)	0.312
Pyoderma gangrenosum	6 (3.3)	13 (1.5)	0.115
Arthritis/arthralgia	42 (22.8)	305 (36.1)	0.001
PSC	15 (8.2)	25 (3)	0.001
Uveitis/iritis	5 (2.7)	52 (6.2)	0.065
Ankylosing spondylitis/sacroiliitis	4 (2.2)	43 (5.1)	0.087
Any EIM	70 (38)	368 (43.5)	0.175

**Table 4:** Therapies ever applied, stratified according to pediatric or adult UC diagnosis

Drugs ever applied	Pediatric UC diagnosis (<18 years old) (*n* = 184), *n* (%)	Adult UC diagnosis (≥18 years old) (*n* = 846), *n* (%)	*p* value
Oral 5-ASA	170 (92.4)	756 (89.4)	0.216
Topical 5-ASA	86 (46.7)	555 (65.6)	<0.001
Any 5-ASA	176 (95.7)	815 (96.3)	0.660
Systemic steroids	133 (72.3)	624 (73.8)	0.681
Topical steroids (enema)	51 (27.7)	297 (35.1)	0.055
Any steroids	147 (79.9)	689 (81.4)	0.626
Azathioprine/mercaptopurine	114 (62)	520 (61.5)	0.901
Methotrexate	29 (15.8)	76 (9)	0.006
Tacrolimus and cyclosporine	19 (10.3)	88 (10.4)	0.976
Anti-TNF (IFX, ADA, and GOL)	78 (42.4)	298 (35.2)	0.067
Vedolizumab	20 (10.9)	93 (11)	0.961
Ustekinumab	0	1 (0.1)	0.641
Tofacitinib	1 (0.5)	1 (0.1)	0.235

ADA, adalimumab; IFX, infliximab; GOL, golimumab; 5-ASA, 5-aminosalicylates.

**Table 5:** Rate of complications at UC diagnosis (% and 95% confidence interval) stratified according to age at disease onset and length of diagnostic delay

Outcome	Short delay adult	Long delay adult Short delay pediatric	Long delay pediatric	p value short adult versus long adult	p value short ped. versus long ped	p value ped. versus adult
Colectomy	3.9 (2.4–5.4)	4.7 (1.9–7.6)	5.1 (1.4–8.7)	4.3 (0–10.2)	0.612	0.844	0.647
UC-related hospitalization	18.0 (15.0–20.9)	17.5 (12.4–22.7)	29.0 (21.4–36.6)	19.6 (8.1–31.0)	0.891	0.211	0.006
Colon dysplasia	0.9 (0.2–1.7)	0.5 (0–1.4)	1.4 (0–3.4)	0 (0–7.7)	0.513	0.412	0.732
Colorectal cancer	0.5 (0–1.0)	0.9 (0–2.3)	0 (0–2.7)	0 (0–7.7)	0.435	1.000	0.296
Any complication (colectomy, hospitalization, dysplasia, or CRC)	21.6 (18.4–24.8)	21.3 (15.8–26.9)	32.6 (24.8–40.4)	23.9 (11.6–36.2)	0.940	0.267	0.009
All EIMs	23.8 (20.5–27.1)	31.8 (25.5–38.0)	21.7 (14.9–28.6)	19.6 (8.1–31.0)	0.022	0.755	0.194
Oral aphthous ulcers	3.3 (1.9–4.7)	3.3 (0.9–5.7)	5.8 (1.9–9.7)	4.3 (0.0–10.2)	0.994	0.707	0.166
Erythema nodosum	2.4 (1.2–3.5)	2.8 (0.6–5.1)	0.7 (0.0–2.1)	0	0.697	0.563	0.099
Pyoderma gangrenosum	0.9 (0.2–1.7)	1.9 (0.1–3.7)	2.9 (0.1–5.7)	0	0.268	0.243	0.292
Arthritis/arthralgia	17.2 (14.2–20.1)	23.7 (18.0–29.4)	8.7 (4.0–13.4)	10.9 (1.9–19.9)	0.035	0.659	0.002
PSC	2.0 (0.9–3.1)	3.3 (0.9–5.7)	6.5 (2.4–10.6)	2.2 (0.0–6.4)	0.293	0.260	0.025
Uveitis/iritis	2.4 (1.2–3.5)	3.8 (1.2–6.4)	0.7 (0.0–2.1)	2.2 (0.0–6.4)	0.269	0.412	0.192
Ankylosing spondylitis/sacroiliitis	2.2 (1.1–3.3)	2.4 (0.3–4.4)	0.7 (0.0–2.1)	2.2 (0.0–6.4)	0.889	0.412	0.313

**Table 6:** Hazard ratios (95% CI; *p* value) associated with decimal logarithm of diagnostic delay in Cox models for various complications (* adjustment is done for age and sex)

Outcome	Pediatric (crude HR)	Pediatric (adjusted* HR)	Adult (crude HR)	Adult (adjusted* HR)
Colectomy	0.038 (0.000–3.301;0.151)	0.043 (0.000–3.816; 0.169)	0.864 (0.489–1.527; 0.615)	0.869 (0.486–1.553; 0.636)
UC-related hospitalization	0.385 (0.134–1.108; 0.077)	0.379 (0.129–1.112; 0.077)	0.751 (0.466–1.213; 0.242)	0.774 (0.479–1.252; 0.297)
Colon dysplasia	NA	NA	3.119 (1.167–8.336; **0.023)**	2.948 (1.158–7.504; **0.023)**
Colorectal cancer	NA	NA	2.785 (0.613–12.657; 0.185)	2.480 (0.626–9.826; 0.196)
Any complication (colectomy, UC-related hospitalization, colon dysplasia, and cancer)	0.349 (0.120–1.009; 0.052)	0.344 (0.117–1.012; 0.053)	1.046 (0.727–1.503; 0.810)	1.072 (0.744–1.543; 0.710)
EIMs (any)	1.441 (0.935–2.222; 0.098)	1.455 (0.939–2.256; 0.094)	1.494 (1.290–1.729; **<0.001)**	1.491 (1.289–1.724; **<0.001)**
Oral erosions/ulcerations	1.213 (0.399–3.682; 0.733)	1.254 (0.407–3.868; 0.694)	1.216 (0.683–2.165; 0.506)	1.212 (0.679–2.162; 0.516)
Erythema nodosum	0.261 (0.018–3.717;0.321)	0.307 (0.027–3.508; 0.342)	2.089 (0.846–5.156; 0.110)	2.088 (0.847–5.146; 0.110)
Pyoderma gangrenosum	1.093 (0.127–9.442; 0.935)	1.116 (0.145–8.558; 0.916)	1.678 (0.204–13.782; 0.630)	1.434 (0.138–14.917; 0.763)
Arthritis/arthralgias	2.048 (1.158–3.622; **0.014)**	1.989 (1.131–3.498; **0.017)**	1.426 (1.210–1.682; **<0.001)**	1.420 (1.204–1.674; **<0.001)**
Primary sclerosing cholangitis	0.924 (0.378–2.257; 0.863)	0.914 (0.320–2.613; 0.866)	1.428 (0.860–2.370; 0.168)	1.542 (0.931–2.555; 0.093)
Uveitis/iritis	3.134 (0.268–36.682; 0.363)	3.563 (0.259–49.049; 0.342)	1.392 (0.785–2.470; 0.258)	1.392 (0.800–2.424; 0.242)
Ankylosing spondylitis/sacroiliitis	1.409 (0.180–11.051; 0.744)	1.478 (0.175–12.511;0.720)	3.404 (2.226–5.205; **<0.001)**	3.243 (2.154–4.882; **<0.001)**

## Notes

Alain M. Schoepfer and Vu Dang Chau Tran contributed equally to this work.
