# Supplementary figures and images for: Impact of Diagnostic Delay on Disease Course in Pediatric- versus Adult-Onset Patients with Ulcerative Colitis: Data from the Swiss IBD Cohort
Source: Inflamm Intest Dis. 2021 Nov 18;7(2):87–96. doi: 10.1159/000520995 (PMC9294935; doi:10.1159/000520995)

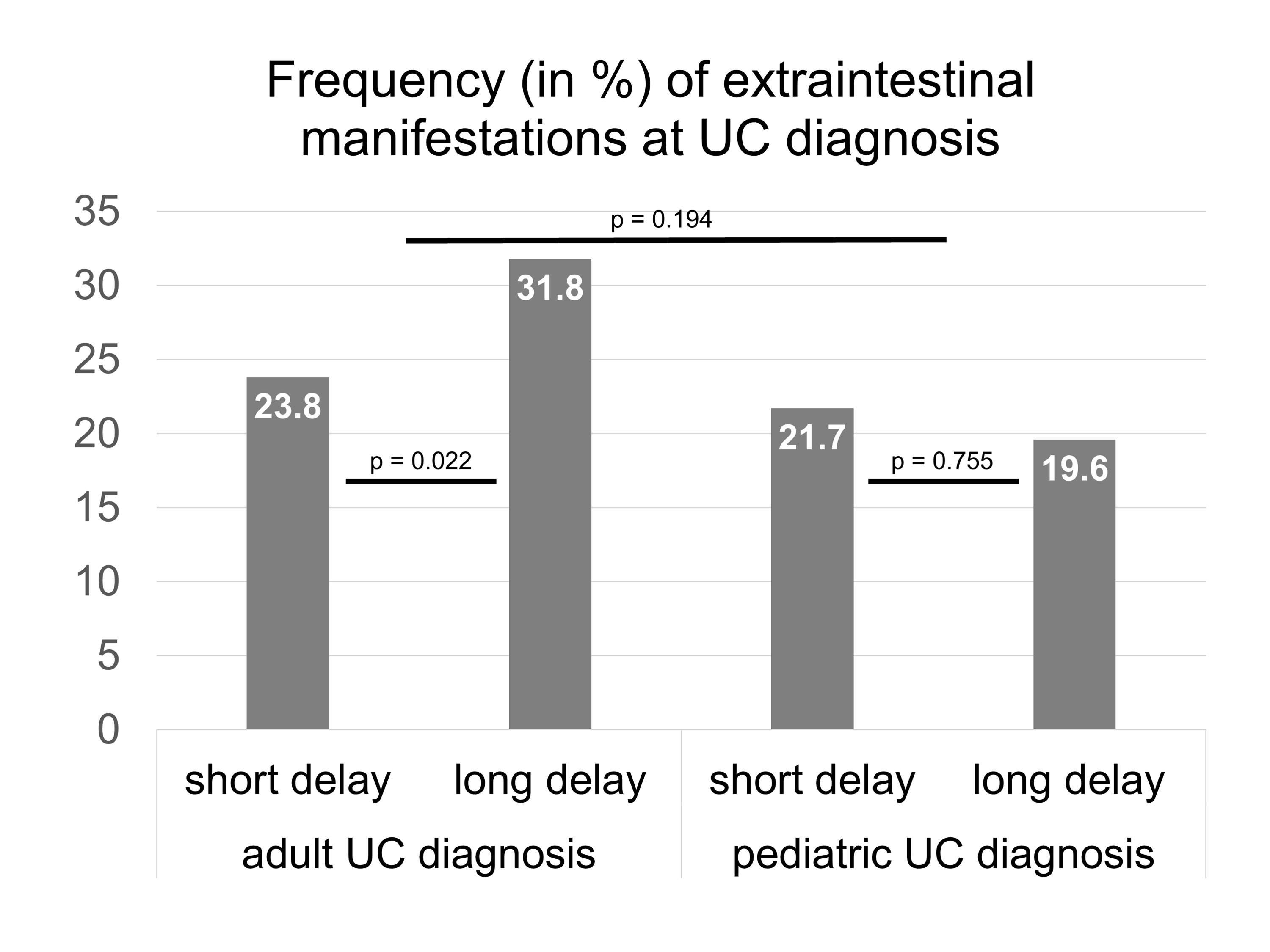

Supplement: Supplementary file 1 — Supplementary data [file iid-0007-0087-s01.tif]

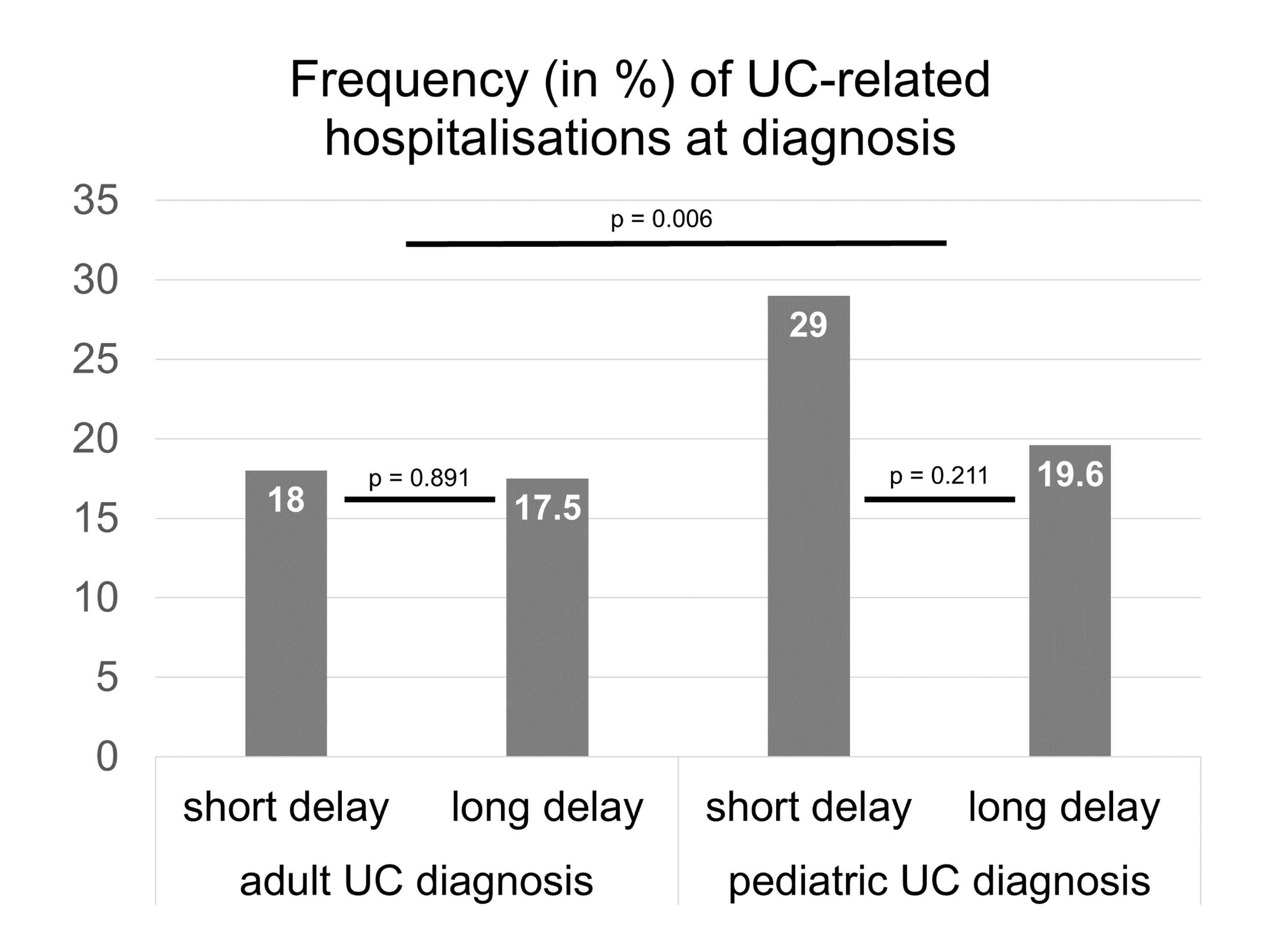

Supplement: Supplementary file 2 — Supplementary data [file iid-0007-0087-s02.tif]
