# Supplementary material for: Impact of Diagnostic Delay on Disease Course in Pediatric- versus Adult-Onset Patients with Ulcerative Colitis: Data from the Swiss IBD Cohort
Source: Inflamm Intest Dis. 2021 Nov 18;7(2):87–96. doi: 10.1159/000520995 (PMC9294935; doi:10.1159/000520995)
Supplement: Supplementary file 3 — Supplementary data [file iid-0007-0087-s03.docx]

**SUPPLEMENTARY FIGURES**

**Supplementary Fig. 1:** Frequency of extraintestinal manifestation at UC diagnosis, stratified according to age of UC diagnosis and length of diagnostic delay

**Supplementary Fig. 2:** Frequency of UC-related hospitalizations at UC diagnosis, stratified according to age of UC diagnosis and length of diagnostic delay
